# Age-stratified fat-soluble vitamin profiles and exploratory analyses of potential links to disease risk and core autistic symptoms among children with ASD: a propensity score matching case-control study

**DOI:** 10.3389/fpsyt.2026.1892933

**Published:** 2026-08-26

**Authors:** Yingying Xin, Jingbo Liu, Taoying Dai, Ting Xia

**Affiliations:** 1 Department of Child Health Care, Institute of Maternal and Child Health, Wuhan Children’s Hospital (Wuhan Maternal and Child Health Care Hospital), Tongji Medical College, Huazhong University of Science & Technology, Wuhan, China; 2 Clinical Research Center of Child Health Care and Developmental & Behavioral Disorders, Institute of Maternal and Child Health, Wuhan Children’s Hospital (Wuhan Maternal and Child Health Care Hospital), Tongji Medical College, Huazhong University of Science & Technology, Wuhan, China

**Keywords:** autism spectrum disorder, ADOS-2, calibrated severity scores, fat-soluble vitamins, propensity score matching

## Abstract

**Objective:**

To evaluate serum levels of fat-soluble vitamins (vitamin D3 (VD3), vitamin D2 (VD2), vitamin A (VA), total vitamin D (total VD), and vitamin E (VE)) in children with autism spectrum disorder (ASD), to analyze their correlation with autistic symptoms, and provide a theoretical basis for clinical nutritional intervention in ASD.

**Methods:**

A propensity score matching (PSM) case-control study was designed, enrolling 50 children diagnosed with ASD at Wuhan Children’s Hospital from January 2023 to December 2025 and 50 typically developing (TD) children undergoing routine physical examinations. With age, sex, BMI and season of blood sampling as covariates, Analysis of covariance (ANCOVA) was used to analyze the differences in fat-soluble vitamin levels between the ASD and TD groups. Conditional logistic regression was used to analyze the association between fat-soluble vitamin levels and the risk of ASD. Benjamini-Hochberg (BH) false discovery rate (FDR) correction was applied for multiple testing of all the vitamin indicators. Pearson correlation analysis was employed to examine the relationship between vitamin levels and autistic symptoms.

**Results:**

After adjustment, serum levels of VD3 and VE in the ASD group were significantly higher than those in the TD group (*p* < 0.05). Significant age-by-group interactions were observed for VE, VD3, and total VD. Age-stratified ANCOVA analysis showed that the log-transformed VD3 (log VD3) elevation in ASD versus TD children was significant only in the older subgroup and increased with age, whereas no significant group differences were detected for total VD or VE within either age stratum. Our findings revealed no statistically significant association between VD3 levels and ASD risk or core symptoms.

**Conclusion:**

Age-dependent subtype-specific differences of fat-soluble vitamins exist in children with ASD, with higher log VD3 only observed in older autistic patients. Unadjusted analyses showed tentative correlations between VD3 and ASD risk as well as repetitive behaviors, which disappeared after FDR correction. Clinically, VD3 and total VD should be assessed separately by age for autistic children. Routine VD screening, with longitudinal monitoring tailored to age, is recommended to guide personalized nutritional management.

## 1 Introduction

Autism spectrum disorder (ASD) is a neurodevelopmental disorder characterized by persistent deficits in social communication/interaction, as well as restricted, repetitive patterns of behavior or interests. According to the latest epidemiological data from the U.S. Centers for Disease Control and Prevention—based on 2022 surveillance data published in April 2025—approximately 1 in 31 children (3.2%) in the United States is diagnosed with ASD (1, 2). Currently, the etiology and pathogenesis of ASD remain incompletely understood. Existing research suggests that ASD is a multifactorial condition, and its onset may be closely related to genetic factors, environmental factors, and their interactions (3). Among the various environmental factors, nutritional status and micronutrient (including vitamins and trace elements) imbalances are particularly noteworthy (4, 5).

Vitamin A (VA) plays a key role in brain development via its active metabolite retinoic acid (RA), which regulates neuronal differentiation, synaptic plasticity, and tissue patterning through retinoic acid receptors (RAR) (6). Studies suggest that VA may improve social ability in ASD by increasing oxytocin levels via the RA-RAR and CD38-oxytocin pathways (7, 8). However, current research on the association between VA and core ASD symptoms remains relatively scarce.

Vitamin D (VD) is a fat-soluble vitamin that exists in two major forms: vitamin D2 (VD2) (ergocalciferol), which is photochemically synthesized in plants, and vitamin D3 (VD3) (cholecalciferol), which is endogenously produced in the skin upon sun exposure or obtained from dietary sources. Both forms undergo 25-hydroxylation in the liver to generate 25-hydroxyvitamin D (25(OH)D), the major circulating metabolite, followed by 1α-hydroxylation in the kidneys to produce the biologically active hormone 1,25-dihydroxyvitamin D (1,25(OH)_2_D), which exerts its effects by binding to the vitamin D receptor (VDR) (9, 10). Studies have shown decreased VD levels in children with ASD, as well as an increased risk of ASD in offspring associated with VD insufficiency during pregnancy (11, 12). Moreover, the potential biological mechanisms regarding the involvement of VD in the pathogenesis of ASD are also being continuously revealed (13, 14). However, VD levels exhibit significant seasonal fluctuations, as sunlight exposure and outdoor activity directly influenced endogenous VD synthesis. Most previous studies examining the correlation between VD and ASD symptoms failed to adjust for the season of blood sampling as a key confounding factor, potentially biasing the effect estimates.

Vitamin E (VE) maintains the structural integrity of cell and subcellular (e.g., vascular) membranes, making it essential for the normal function of the reproductive, circulatory, nervous, immune, and muscular systems (15). Previous studies have reported that VE concentrations in ASD are reduced, and this change is associated with ASD-like behaviors (16). Overall, evidence linking VE to ASD is very limited, mostly derived from animal models (17, 18).

Oxidative stress is recognized as a core mechanism underlying ASD pathophysiology, characterized by an imbalance between excessive reactive oxygen species(ROS) production and antioxidant defenses, which leads to oxidative damage to lipids, proteins, and DNA in neurons and disrupts neurodevelopment (19). VA, VD, and VE, although acting through distinct pathways, all converge on this oxidative stress network: RA exerts protective effects by scavenging free radicals, enhancing antioxidant enzyme activity, and reducing ROS production, whereas VA deficiency exacerbates oxidative stress by impairing thioredoxin and glutathione (GSH) synthesis (20). 1,25(OH)_2_D activates VDR, regulating antioxidant enzyme expression, promoting GSH synthesis, and inhibiting ROS production in astrocytes (21). VE directly scavenges lipid peroxyl radicals via its phenolic hydroxyl group, thereby terminating the chain reaction of lipid peroxidation and protecting neuronal membranes (22). These three vitamins act on complementary targets within the oxidative stress pathway—free radical scavenging (VA), transcriptional regulation of antioxidant enzymes and GSH (VD), and chain-breaking termination of lipid peroxidation (VE)—and together constitute a multilayered defense system against oxidative damage in ASD.

However, whether VD metabolism in ASD exhibits subtype-specific (VD3 vs. total VD) and age-dependent patterns remains unclear, as most previous studies have relied on single-time-point total VD measurements without age stratification. To address these gaps, the present study is designed to (1) compare serum levels fat-soluble vitamins between ASD and typically developing (TD) groups across different age strata (2), evaluate the associations between these vitamin levels and the risk of ASD, and (3) examine their relationship with core symptom severity, with the ultimate aim of refining clinical monitoring and stratification strategies. This work may inform future investigations by emphasizing the need for age-stratified and subtype-differentiated analytical approaches.

## 2 Methods

### 2.1 Ethical approval of the study protocol

This study is a retrospective propensity score matching (PSM) case-control study that analyzed data collected from children during routine clinical practice, without any intervention involving the study participants. The research protocol was reviewed and approved by the Medical Ethics Committee of Wuhan Children’s Hospital (Approval No.2020R112-E03). All patient data were anonymized prior to analysis, and strict confidentiality principles were observed. This study was conducted in accordance with the Declaration of Helsinki and relevant national ethical review regulations.

### 2.2 Experimental subjects and TD children

This retrospective observational study employed PSM to balance baseline characteristics between children with ASD and TD controls. Propensity scores were calculated using logistic regression with case-control status as the dependent variable and age, sex, BMI and season of sampling. A 1:1 nearest neighbor matching with a caliper of 0.02 was applied. Post-matching balance tests revealed no significant differences in any covariate (SMD < 0.2), indicating good matching quality. Ultimately, 50 ASD children and 50 TD children were included in the analysis.

The ASD group were diagnosed at department of Child Health Care of Wuhan Children’s Hospital between January 2023 and December 2025. All enrolled participants underwent a series of systematic examinations, including diagnostic hearing tests, blood amino acid analysis, electroencephalography (EEG), cranial magnetic resonance imaging (MRI), thyroid function tests, and chromosomal analysis, to exclude hearing impairment, epilepsy, hypothyroidism, inherited metabolic disorders, and other organic diseases. The diagnosis of ASD further ruled out cases with global developmental delay, intellectual disability, developmental language disorder, reactive attachment disorder, and known syndromic autism. Additionally, the Gesell Developmental Scale, Wechsler Intelligence Scale, and Achenbach Child Behavior Checklist were administered to assess the presence of any comorbidities.

TD children were selected from those undergoing routine health check-ups at our hospital during the same period. The inclusion criteria for the TD group were as follows: (1) Chinese nationality; (2) normal hearing and vision; and (3) scores above 85 in all domains of the China Developmental Scale for Children Aged 0–6 Years (CDSC), as assessed by CDSC-certified nurses. The exclusion criteria included: (1) neoplastic diseases; (2) acute infectious diseases; and (3) neurological or psychiatric disorders (e.g., developmental delay, intellectual disability, epilepsy, etc.), as well as a history of medication use within two weeks prior to enrollment.

The inclusion criteria for children with ASD were as follows: (1) meeting the diagnostic criteria of the Diagnostic and Statistical Manual of Mental Disorders, Fifth Edition (DSM-5) (23), with the diagnosis confirmed jointly by at least two associate chief physicians; (2) administration of the Autism Diagnostic Observation Schedule, Second Edition (ADOS-2) by a certified assessor, with a Calibrated Severity Score (CSS) ≥ 4; (3) no use of any medication within two weeks prior to enrollment; and (4) normal diagnostic hearing examination. The exclusion criteria included: (1) syndromic autism (e.g., fragile X syndrome, Rett syndrome, etc.); and (2) inherited metabolic diseases (e.g., hypothyroidism).

Five fat-soluble vitamins are not routinely measured in children with ASD and TD groups. The TD group data in this study were not derived from research-specific testing, but rather from a nutritional and metabolic screening database of the Health Examination Center in our hospital. During the annual health check-ups at this center, parents could choose a personalized examination package that included fat-soluble vitamin profiling (including VA, VD, VE, etc.), and the test results were routinely recorded in the electronic medical record system.

### 2.3 Laboratory measurements

The serum concentrations of fat-soluble vitamins (VA, VD2, VD3, total VD, and VE) in all enrolled children were determined by ultra-high performance liquid chromatography (UPLC) at the Eugenic Genetics Laboratory of our hospital. The certified clinical laboratory participates in regular internal and external quality control programs, and intra-assay and inter-assay coefficients of variation were less than 10% for all vitamin measurements. The diagnostic criteria were as follows: serum VA concentration < 300 ng/mL was classified as deficiency, and 300–800 ng/mL as normal; VD2 concentration < 1.2 ng/mL as deficiency, and 1.2-3.5 ng/mL as normal; total VD concentration < 30 ng/mL as insufficiency, and 30–100 ng/mL as normal; VE concentration < 5168.6 ng/mL as deficiency, 5168.6-20,000 ng/mL as normal, and >20,000 ng/mL as excess.

### 2.4 Diagnosis by ADOS-2

The ADOS-2 was used to conduct a structured assessment of autism-related behaviors in order to measure the severity of autistic symptoms. ADOS-2 includes five modules (Toddler Module and Modules 1 to 4), selected based on the child’s age and language ability, to evaluate ASD-related symptoms across domains such as social affect, play and creativity, and restricted and repetitive behaviors (24). The ADOS-2 CSS provides a quantitative assessment of ASD symptom severity, allowing comparability of symptoms across children of different ages and language abilities, with the following rating levels: scores of 1–2 indicate “minimal or no evidence”, 3-4 “low”, 5-7 “moderate” and 8-10 “high”; a score of 4 or above meets the diagnostic criteria for ASD. The ADOS-2 CSS for social affect (SA-CSS) and restricted and repetitive behaviors (RRB-CSS) were specifically designed to provide domain-specific estimates of ASD symptom severity that are relatively independent of child characteristics such as age and language level (25).

### 2.5 Statistical analysis

Statistical analysis was performed using IBM SPSS Statistics software (version 27.0; IBM Corp., Armonk, NY, USA). GraphPad Prism Version 10.0 was used for graphics. First, to control for potential confounding bias, PSM was used to match the ASD group and the TD group at a 1:1 ratio based on variables such as age, sex, BMI and season of blood sampling with a caliper value set at 0.02 (refers to the raw PS probability scale ranging from 0 to 1). Before and after PSM, between-group differences in baseline characteristics were evaluated using both significance testing and standardized mean difference (SMD). SMD, which is independent of sample size, was used as the primary metric to assess covariate balance. An absolute SMD <0.1 was defined as excellent intergroup balance, 0.1 ≤ SMD <0.2 as acceptable balance, and SMD ≥0.2 as poor balance. Normality testing (Shapiro-Wilk test) and homogeneity of variance testing were conducted for continuous variables. Continuous data following a normal distribution were expressed as mean ± standard deviation (mean ± SD), and between-group comparisons were performed using independent-sample t-test. Continuous data not following a normal distribution were expressed as median (25^th^, 75^th^), and between-group comparisons were performed using the Mann-Whitney U test. Sample testing time was classified as two categories: spring/summer (high sunlight exposure season) and autumn/winter (low sunlight exposure season).

Prior to analysis, vitamin data with skewed distribution were log-transformed to meet the normality and homogeneity of variance assumptions of parametric statistics. Analysis of covariance (ANCOVA) was applied to compare group differences, with age, sex, BMI and season of sampling time included as covariates. A 1:1 matched conditional logistic regression model was used to evaluate the association between fat-soluble vitamin levels and ASD risk, with results expressed as odds ratios (*OR*s) and 95% confidence intervals (*CI*s). Primary conditional logistic regression models were adjusted for age and sex, as 0.1 ≤ SMD <0.2 indicated suboptimal balance after PSM. These two variables were thus incorporated as confounding covariates. For sensitivity analysis, BMI was further added to the fully adjusted model based on the primary adjustment framework. The sensitivity analysis was employed to evaluate the possible occurrence of overfitting and to confirm the stability of the primary results under multiple adjustments. Pearson correlation analysis was used to analyze the correlations between fat-soluble vitamin levels and clinical symptoms in children with ASD. To correct for multiple comparisons, we applied the Benjamini-Hochberg (BH) procedure to estimate the False Discovery Rate (FDR). FDR-adjusted *P* value < 0.05 was considered statistically significant. All tests were two-tailed, and a *P*-value < 0.05 was considered statistically significant.

## 3 Results

### 3.1 Study population

In this study, a total of 65 ASD patients were initially collected. Fifteen patients were excluded due to missing key data (ADOS-2), leaving 50 ASD children for inclusion. Concurrently, information from 561 healthy controls was collected. Given that there were statistically significant differences in baseline characteristics between the ASD and TD groups, PSM was performed to select control participants based on key covariates. Before PSM, there were significant imbalances in age (SMD = 0.759), sex (SMD = 0.732), BMI (SMD = 0.087), and season of blood sampling (SMD = 0.202) distribution, with SMD values exceeding 0.2, indicating notable baseline differences between the ASD and TD groups. After PSM, a total of 50 pairs of ASD and TD children with balanced baseline characteristics were obtained. The imbalances were substantially eliminated. The SMD decreased to 0.146 for sex, 0.107 for age, 0.039 for BMI and 0.082 for season of blood sampling. Among the 50 ASD participants, according to the classification criteria of the ADOS-CSS, 10 (20%) were classified as mild ASD, 36 (72%) as moderate ASD, and 4 (8%) as severe ASD. The baseline characteristics of the participants in both groups are detailed in **Table 1**.

**Table 1:** Basic information of ASD and TD children before and after PSM.

Characteristics	Before PSM			After PSM		
	ASD (n=50)	TD (n=561)	*SMD*	ASD (n=50)	TD (n=50)	*SMD*
Sex n (%)						
Male (n)	45 (90.0%)	337 (60.1%)	0.732	45 (90.0%)	47 (94.0%)	0.146
Female (n)	5 (10.0%)	224 (39.9%)		5 (10.0%)	3 (6.0%)	
Age years median (25^th^, 75^th^)	3.15 (2.30, 4.83)	5.46 (2.74, 8.30)	0.759	3.15 (2.30, 4.83)	3.18 (2.14, 4.98)	0.107
BMI median (25^th^, 75^th^)	15.77 (14.84, 16.53)	15.33 (14.32, 16.44)	0.087	15.77 (14.84, 16.53)	15.52 (14.81, 16.48)	0.039
Sampling season						
Spring & summer (n)	30 (60.0%)	390 (69.5%)	0.202	30 (60.0%)	32 (64.0%)	0.082
Autumn & winter (n)	20 (40.0%)	171 (30.5%)		20 (40.0%)	18 (36.0%)	
CARS median (25^th^, 75^th^)	30.5 (28.5, 33)	—		30.5 (28.5, 33)	—	
ADOS-2 CSS median (25^th^, 75^th^)	6 (5, 7)	—		6 (5, 7)	—	
SA-CSS median (25^th^, 75^th^)	5 (1, 6)	—		5 (1, 6)	—	
RRB-CSS median (25^th^, 75^th^)	7 (6, 8)	—		7 (6, 8)	—	

Continuous variables with normal distribution are presented as mean ± standard deviation; non-normally distributed continuous variables are presented as median (25^th^, 75^th^). Categorical variables are shown as n (%). CARS, ADOS-2, SA-CSS and RRB-CSS assessments were only performed in children with ASD. *P* < 0.05 indicates statistical significance.

### 3.2 Age-by-group interactions and age-stratified ANCOVA analyses of fat-soluble vitamins

Normality testing showed that VA and VE concentrations followed a normal distribution in both groups, whereas VD2, VD3, and total VD concentrations exhibited a skewed distribution. Crude between-group comparison showed statistically significant differences in VD3 between the two groups. VD2, VD3, and total VD were log-transformed. The ANCOVA results in **Table 2** showed that after adjusting for covariates age, sex, BMI and season of sampling, serum levels of VD3 and VE were significantly higher in the ASD group than in the TD group (FDR-adjusted *P* < 0.05). Given that age, sex, and BMI may modulate the differences in fat-soluble vitamin levels between the two groups, interaction terms (group × age, group × sex, and group × BMI) were incorporated into the ANCOVA models. When log VD3, log (total VD), and VE served as dependent variables, the group × age interaction was found to be significant (*F* = 5.648, *P* < 0.001), indicating that the differences in VD3, total VD, and VE levels between the ASD and TD groups varied with age. To further explore this age-dependent effect, subgroup ANCOVA was performed by stratifying the participants according to the median age into lower- and higher-age subgroups, with adjustment for sex, BMI, and season of blood collection. In the higher-age subgroup, the ASD group exhibited significantly higher log-transformed VD3 (log VD3) levels (1.657 ± 0.117) compared with the TD controls (1.458 ± 0.146) (*F* = 9.425, FDR-adjusted *P* < 0.001). These findings suggest that the disparity in serum VD3 levels between ASD and TD children is modulated by age. No significant group differences were detected for total VD or VE within either age stratum. The results were shown in **Table 3**.

**Table 2:** ANCOVA analysis of fat-soluble vitamin levels between the ASD group and the PSM-matched TD group after adjusting for age, sex, BMI and season of sampling.

Levels of fat- soluble vitamins (ng/ml)	ASD group (n=50)	TD group (n=50)	Adjusted *F* value	Adjusted *P* value	FDR-adjusted *P* value
LogVD3	1.626 ± 0.116	1.547 ± 0.153	7.433	<0.001***	<0.001***
LogVD2	-0.146 ± 0.605	-0.017 ± 0.633	0.595	0.704	0.704
Log total VD	1.540 ± 0.140	1.575 ± 0.150	1.800	0.122	0.203
VA	370.896 ± 81.113	378.517 ± 76.530	1.135	0.347	0.434
VE	9431.263 ± 2548.367	9199.737 ± 2531.688	2.680	0.019*	0.048*

Data of skewed distributed vitamins (VD3, VD2, total VD) were log-transformed to approximate normal distribution prior to ANCOVA. ANCOVA was used to compare fat-soluble vitamin levels between groups, adjusting for age, sex, BMI and season of sampling. Data are presented as mean ± SD. ASD, autism spectrum disorder; TD, typically developing children. All above *P*-values were adjusted for multiple comparisons using the Benjamini–Hochberg false discovery rate (FDR) procedure. *p < 0.05, **0.05< p < 0.01, ***0.01 < p < 0.001.

**Table 3:** Age-stratified ANCOVA subgroup analysis results for fat-soluble vitamin level differences between ASD and TD groups.

Levels of fat- soluble vitamins (ng/ml)	ASD group (n=25)	TD group (n=25)	Adjusted *F* value	Adjusted *P* value	*FDR*-adjusted *P* value
LogVD3					
Lower-age	1.595 ± 0.108	1.635 ± 0.102	1.895	0.115	0.344
Higher-age	1.657 ± 0.117	1.458 ± 0.146	9.425	<0.001***	<0.001***
Log total VD					
Lower-age	1.618 ± 0.098	1.663 ± 0.107	1.394	0.245	0.368
Higher-age	1.463 ± 0.133	1.487 ± 0.136	2.046	0.091	0.136
VE					
Lower-age	10355.547 ± 2515.035	10104.166 ± 2545.621	1.077	0.386	0.386
Higher-age	8506.980 ± 2270.187	8295.307 ± 2213.959	0.627	0.680	0.680

Significant group-by-age interaction terms were identified for log-transformed VD_3_, log-transformed total VD and VE in the overall cohort. Participants were stratified into younger and older age subgroups based on the median age cutoff (<3.145 vs ≥3.145). ANCOVA was conducted within each stratum, adjusting for confounding covariates including sex, blood sampling season, age and BMI. All above *P*-values were adjusted for multiple comparisons using the Benjamini–Hochberg false discovery rate (FDR) procedure. *p < 0.05, **p < 0.01, ***p < 0.001.

### 3.3 Exploratory analyses of the association between VD3 Levels and ASD risk

After adjusting for age and sex, conditional logistic regression analysis showed a nominal positive association between VD3 levels and ASD risk (OR = 1.045, 95% CI: 1.006 - 1.086, raw *P* = 0.023, FDR-adjusted *P* = 0.081). No statistically significant correlations were found between VD2, total VD, VA or VE levels and ASD risk in this study. Sensitivity analysis via multivariable-adjusted conditional logistic regression was performed with additional adjustment for sex, age and BMI. sex, age and BMI demonstrated no statistically significant association with ASD risk in children (all FDR-adjusted *P >*0.05). For VD3, the regression coefficient changed slightly to 0.046, with raw *P* = 0.023 remaining statistically significant, FDR-adjusted *P* value 0.081, above the significance cutoff. The direction and magnitude of the VD3-ASD association had no substantial shift relative to the primary model. Detailed results are shown in **Table 4** and **Supplementary Table 1**.

**Table 4:** conditional logistic regression analysis of VD3 level and the risk of ASD in children.

Variables	B	OR (95%*CI*)	*P* value	*FDR-P value*
VD3	0.044	1.045 (1.006 - 1.086)	0.023*	0.081
Age	0.262	1.300 (0.661 - 2.555)	0.447	0.451
Sex	-1.931	0.145 (0.001 - 17.007)	0.427	0.451

B, regression coefficient; OR, odds ratio; CI, confidence interval. The model was adjusted for age and sex. *Raw *P* < 0.05; FDR-adjusted *P* value was calculated to account for multiple comparisons.

### 3.4 Correlation analysis of fat-soluble vitamins with clinical symptoms of ASD

Pearson correlation analysis examined the associations between serum vitamin levels (VA, log VD2, log VD3, log (total VD), and VE) and ADOS-2 CSS as well as its core symptom dimensions (RRB-CSS and SA-CSS). The results revealed no statistically significant correlations between the levels of fat-soluble vitamins and ADOS-2 CSS or SA-CSS. Regarding restricted and repetitive behaviors, VA, VD2, VE, and VD levels also showed no statistically significant associations with RRB-CSS. However, logVD3 showed a weak negative raw correlation with RRB-CSS (*r*=-0.302, raw *P*=0.033), suggesting that lower VD3 levels are associated with more severe stereotyped behaviors, yet the association lost statistical significance after FDR adjustment (*P* = 0.459). The results were shown in **Table 5**.

**Table 5:** Correlation between fat-soluble vitamin levels and RRB-CSS in children with.

Variables	*r* value	*P* value	*FDR*-adjusted *P* value
Log VD3	-0.302	0.033*	0.459
Log VD2	0.000	0.998	0.998
Log (total VD)	-0.118	0.4127	0.998
VA	-0.200	0.1631	0.612
VE	0.003	0.983	0.998

Pearson correlation was used for the associations between fat-soluble vitamins (VD3, VD2, total VD, VA, VE) and stereotypic behavior score (Data of skewed distributed vitamins were log-transformed to approximate normal distribution). All above *P*-values were adjusted for multiple comparisons using the Benjamini–Hochberg false discovery rate (FDR) procedure. *refers to Raw *P* < 0.05.

## 4 Discussion

This 1:1 PSM case-control study systematically investigated intergroup disparities in serum fat-soluble vitamins between ASD and TD children, as well as their correlations with ASD risk and core behavioral symptoms. After adjusting for age, sex, BMI and sampling season, ANCOVA revealed significantly higher logVD3 and VE in ASD children, with robust differences retained after FDR correction. Significant group-by-age interaction was detected, and stratified analysis confirmed elevated VD3 in ASD only among older children, indicating age modifies vitamin discrepancies across groups. After FDR correction, fat-soluble vitamin levels showed no statistically significant association with ASD risk or core symptoms.

In the simple two-group comparison without adjusting for confounding factors, only logVD3 was significantly higher in the ASD group than in the TD group. However, after adjusting for age, sex, BMI, and season of blood collection in an ANCOVA model, both logVD3 and VE remained significantly elevated in the ASD group (FDR*-*adjusted *P* < 0.05). The above results indicate that the four variables exerted a considerable confounding effect on VE levels, thereby masking the true between-group difference in VE between the ASD and TD groups. The findings are somewhat inconsistent with the conclusions of most previous studies (26, 27). To reconcile this discrepancy, several factors warrant consideration. First, seasonal variation, a well-established confounder, was carefully controlled in our multivariate models, excluding its potential contribution to the observed elevation (28). Second, genetic heterogeneity may play a role. A previous study on VDR gene polymorphisms reported that children with ASD had significantly higher serum 25(OH)D levels than controls, and that the FokI polymorphism significantly influenced this parameter (29), suggesting that genetic variants may modulate interindividual differences in VD homeostasis in ASD. Third, and perhaps most directly, dietary and supplement practices may account for the higher levels observed in our ASD cohort. Up to 56% of children with ASD use dietary supplements, predominantly multivitamins and minerals, and many exhibit abnormal eating behaviors and strong food selectivity (30, 31). These behavioral patterns could lead to disproportionately high intake of fat-soluble vitamins via fortified foods or supplements. This interpretation is supported by Zhang et al., who found significantly elevated plasma levels of vitamin B1, nicotinamide, pyridoxamine dihydrochloride, and VE in boys with ASD (which were not in the deficiency range) (32). Similarly, a case-control study reported that dietary VE intake in children with ASD was significantly higher than that in the control group (33). Nevertheless, it should be acknowledged that we did not collect detailed dietary intake data or quantify the frequency and dosage of supplement use, which precludes direct confirmation of the contribution of these behavioral factors to the observed vitamin elevations. Furthermore, we did not assess individual sunlight exposure or outdoor activity time, both of which are key determinants of endogenous VD3 synthesis. Although we adjusted for season of blood sampling as a proxy for ambient UV radiation, this adjustment cannot fully capture individual-level variability in sun exposure behaviors. Collectively, while our results diverge from the prevailing literature on VD, the combination of genetic polymorphisms, atypical dietary patterns, and widespread supplement use may offer a plausible explanation, but these interpretations require direct validation in future studies incorporating detailed dietary records, supplement use histories, and objective measures of sunlight exposure.

Our age-stratified ANCOVA analysis further revealed that the elevation of logVD3 in children with ASD, relative to TD controls, reached statistical significance only in the higher-age subgroup, and this group difference widened with increasing age, suggesting that age acts as an important effect modifier in the relationship between ASD and VD3 status. Previous studies have confirmed that VD metabolism exhibits age-related heterogeneity in children with ASD. Within the ASD population, Şengenç et al. reported that 25(OH)D levels in adolescents aged 11–18 years were significantly lower than in younger children (< 11 years), with approximately 95% of the cohort being VD deficient or insufficient (34). Similarly, Shan et al. found a significant negative association between age and VD concentration, and noted that VD levels were negatively correlated with electronic screen time and positively correlated with sunlight exposure (35). These studies collectively indicate an age-related decline in VD within the ASD population. However, a 2026 large cross-sectional study (n=14,911) reported that, after adjusting for age, sex, and season, fat-soluble vitamin levels in the ASD group were largely comparable to controls, suggesting no consistent deviation at the population level (36).

We propose that these seemingly discrepant findings may be reconciled within a single framework: within the ASD group, total VD and VE levels decline with age (consistent with Şengenç et al. and Shan et al.), whereas the between-group difference in VD3 (ASD vs. TD) widens with age (as observed in our study). Most early studies only measured total circulating VD without separately quantifying VD3 fractions, which merely captured the overall age-related reduction and failed to identify this unique subtype-specific elevation of VD3. Correspondingly, this age-dependent effect may be underrepresented in large cross-sectional studies that rely on adjustment rather than stratification or interaction analyses. As children with ASD grow older, children with ASD may develop more pronounced aberrant eating behaviors and greater supplement intake, partially counterbalancing reduced endogenous synthesis. Concurrently, age-related behavioral changes—including increased screen time and decreased sunlight exposure, as documented by Shan et al.—may further widen the lifestyle gap between ASD children and their TD peers.

The tentative positive trend between VD3 and ASD risk observed in our study is aligns with a small subset of prior cross-sectional observations. Zou et al. found that higher VD levels were associated with social impairment in children with ASD, suggesting that within the ASD population, higher VD levels are not invariably linked to better clinical outcomes (37). Furthermore, a 2024 Mendelian randomization study concluded that there is no causal relationship between VD and ASD, and that ASD itself may lead to decreased VD levels (38). These published findings are not contradictory to the weak cross-sectional trend noted in our matched cohort. Rather, the nominal correlation derived from cross-sectional case-control data may arise from reverse causation—where ASD-specific lifestyle patterns modify VD3 status—or residual unmeasured confounding factors, rather than proving a direct causal effect of elevated VD3 on ASD risk. Given that the signal for VD3 failed to retain statistical significance after FDR adjustment for multiple comparisons, our results only provide preliminary suggestive clues, and external validation based on larger multicenter cohorts is warranted to verify these observations.

Finally, we examined the relationship between fat-soluble vitamin levels (VA, VD2, VD3, total VD and VE) and ASD symptom severity. Preliminary correlation analyses in the present study identified a nominal negative trend between logVD3 and repetitive stereotyped behaviors, which may offer a novel research direction regarding the regulatory role of VD in core autistic symptoms. Nevertheless, all correlation signals lost statistical significance following FDR correction for multiple comparisons. One possible explanation is that VD levels may play a role in ASD susceptibility, but once the disorder is established, there may be no simple linear dose–response relationship between circulating VD levels and global symptom severity (39). A 2024 cross-sectional study of 80 children with ASD (aged 2–6 years) further supports our null findings: although 63.8% had VD insufficiency, serum VD levels were not correlated with DSM-5 severity levels or CARS scores (39). This suggests that VD deficiency may act as an environmental factor in genetically susceptible individuals rather than serving as a biomarker that fluctuates with current symptom severity. The absence of statistical significance in correlation analyses can be attributed to our limited sample size and stringent multiple comparison correction, which attenuated weak nominal correlative signals. Furthermore, single-time-point serum vitamin measurement fails to capture long-term cumulative nutritional exposure that modulates autistic behavioral phenotypes. Future large-scale multicenter cohorts incorporating serial repeated vitamin testing are required to stratify and quantify the age- and nutrition-dependent relationships between VD3 and core autistic symptoms.

## 5 Conclusion

In conclusion, our findings reveal age-dependent differentiation in fat-soluble vitamin profiles among children with ASD. Specifically, log-transformed VD3 levels were significantly higher in older children with ASD compared with their age-matched TD peers. Although unadjusted analyses suggested a weak negative correlation between logVD3 and restricted and repetitive behaviors, as well as a positive trend between VD3 levels and ASD risk, none of these correlative signals survived FDR correction for multiple testing. These exploratory findings suggest that routine VD screening and longitudinal monitoring tailored to age, is recommended to guide personalized nutritional management. Importantly, Clinical evaluation must distinguish VD3 from total VD and adopt age-stratified assessment strategies.

Several limitations should be noted: Small sample size and strict FDR correction weakened statistical power and attenuated weak signals. Single-time-point measurements preclude assessment of long-term vitamin exposure. Although matched for key covariates, the cross-sectional design remains susceptible to residual confounding. Future prospective cohorts, Mendelian randomization, and RCTs are required to validate our findings and establish causality.

## Data Availability

The raw data supporting the conclusions of this article will be made available by the authors, without undue reservation.
