# Supplementary material for: Age-stratified fat-soluble vitamin profiles and exploratory analyses of potential links to disease risk and core autistic symptoms among children with ASD: a propensity score matching case-control study
Source: Front Psychiatry. 2026 Aug 26;17:1892933. doi: 10.3389/fpsyt.2026.1892933 (PMC13553793; doi:10.3389/fpsyt.2026.1892933)
Supplement: Supplementary file 1 [file Table1.docx]

**Table S1 conditional logistic regression analysis of VD3 level and the risk of ASD in children (sensitivity analysis)**

| variables | B | *OR (95%CI)* | *P* value | *FDR-*adjusted *P* value |
| --- | --- | --- | --- | --- |
| age | 0.349 | 1.417 (0.695 - 2.891) | 0.337 | 0.451 |
| sex | -2.682 | 0.068 (0.000 -12.450) | 0.312 | 0.451 |
| BMI | 0.120 | 1.127 (0.825 - 1.540) | 0.451 | 0.451 |
| VD3 | 0.046 | 1.047 (1.006 – 1.090) | 0.023* | 0.081 |

Note: B, regression coefficient; OR, odds ratio; CI, confidence interval. The model was adjusted for age, sex and BMI. Raw**P* < 0.05 indicates statistical significance. *FDR*-adjusted *P* value was calculated to account for multiple comparisons. The odds ratios were not shown in this table.
